# Fecal colitis obliterans and cytomegalovirus enteritis after pancreaticoduodenectomy for resectable pancreatic cancer

**DOI:** 10.1007/s12328-025-02219-7

**Published:** 2025-09-12

**Authors:** Kei Nakagawa, Kazuhiro Takami, Hiroto Sakurai, Yuki Yoshino, Kenichiro Yambe, Noriko Kondo, Kuniharu Yamamoto, Morihisa Hirota, Chikashi Shibata, Yu Katayose

**Affiliations:** 1 Division of Hepato-Biliary and Pancreatic Surgery, Tohoku Medical and Pharmaceutical University, 1-15-1 Fukumuro, Miyagino-ku, Sendai, Miyagi 983-8356 Japan; 2 Division of Gastroenterology, Tohoku Medical and Pharmaceutical University, Sendai, Japan; 3 Division of Gastroenterologic Surgery, Tohoku Medical and Pharmaceutical University, Sendai, Japan

**Keywords:** Resectable pancreatic cancer, Neoadjuvant therapy, Colitis obliterans, Cytomegalovirus enteritis

## Abstract

A 74-year-old male patient with a resectable pancreatic cancer underwent radical surgery after receiving neoadjuvant chemotherapy. The patient went into shock after surgery owing to sepsis caused by fecal impaction. The sepsis healed rapidly after the patient was encouraged to defecate through stool evacuation. He subsequently presented with gastrointestinal bleeding from an aneurysm, requiring interventional radiology hemostasis and blood transfusion. Bleeding continued after arterial embolization. On the 35th day after surgery, cytomegalovirus enteritis was detected during a lower gastrointestinal endoscopy, for which the patient was treated with ganciclovir. Pancytopenia and constipation resulting from preoperative treatment likely contributed to the intraoperative progression of colitis obliterans. Colonic damage and gastrointestinal hemorrhage may have led to cytomegalovirus enteritis due to immunocompromised status. The combination of preoperative chemotherapy, massive bleeding, and colitis obliterans is important in an immunocompromised state and can occur in the perioperative period of pancreatic cancer. This case demonstrates the importance of perioperative bowel control during highly invasive surgery with preoperative treatment and colonoscopy at the time of hemorrhage.

**Supplementary Information:**

The online version contains supplementary material available at 10.1007/s12328-025-02219-7.

## Introduction

Pancreatic cancer frequently recurs after resection, even in resectable cases. Postoperative adjuvant chemotherapy has been tested to improve this condition, with evidence of its efficacy [1, 2]. However, the completion rate of adjuvant chemotherapy is reportedly low [3]. Recurrence is also frequently observed, even in cases where adjuvant chemotherapy has been administered. Against this background, clinical trials for preoperative treatment have been actively conducted, with chemotherapy, radiotherapy, and surgical resection being used as a multidisciplinary approach termed total neoadjuvant therapy [4, 5]. Although such multidisciplinary treatment is expected to improve pancreatic cancer outcomes, it may also lead to adverse events and complications, resulting in unexpected conditions.

Here, we report a case of sepsis caused by fecal colitis resulting from intraoperative obstructive colitis, which occurred after radical resection of resectable (R) pancreatic head cancer following preoperative chemotherapy. The patient also had cytomegalovirus (CMV) enteritis following bleeding complications. In both cases, the clinical course, computed tomography (CT) imaging, and colonoscopic findings were important. This case provides valuable insight into the treatment of pancreatic cancer, which requires a multidisciplinary approach and carries a high risk of complications associated with radical resection.

## Case report

The patient was a 74-year-old male with a height of 165.5 cm, weight of 69.5 kg, and body mass index of 25.4 kg/m^2^. His chief complaints were brown-colored urine and anorexia. The patient’s medical history included prostate cancer, hypertension, and diabetes mellitus.

**Fig. 1:**
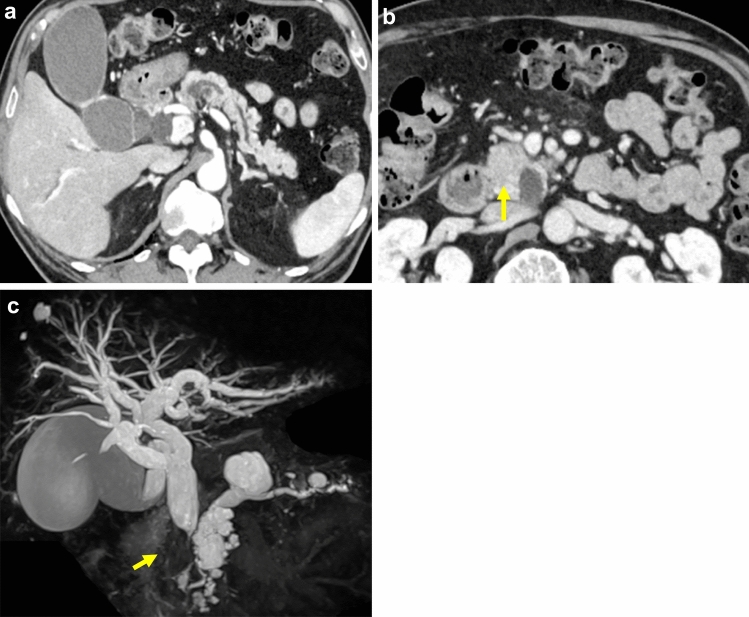
CT and MRCP findings. **a** Dilation of the main pancreatic duct is seen. **b** Tumor of mild low density in the groove region of the pancreatic head (arrow). **c** Stenosis of the bile duct due to a pancreatic tumor (arrow) and branched pancreatic duct-type IPMN. *IPMN* intraductal papillary mucinous neoplasm; *CT* computed tomography; *MRCP* magnetic resonance cholangiopancreatography

He visited his local doctor due to brown urine and anorexia, and was referred to our gastroenterology clinic for a thorough examination of obstructive jaundice. Numerous cystic lesions were identified, primarily in the head and body of the pancreas, along with evidence of a branched pancreatic duct-type intraductal papillary mucinous neoplasm (IPMN) (Fig. 1). Endoscopic retrograde cholangiopancreatography was performed, and a biliary stent was implanted to reduce the yellowish coloration. Endoscopic ultrasound-guided fine needle aspiration was performed on a mass in the pancreatic head, which revealed adenocarcinoma. Furthermore, the diagnosis of pancreatic head cancer was made.

**Fig. 2:**
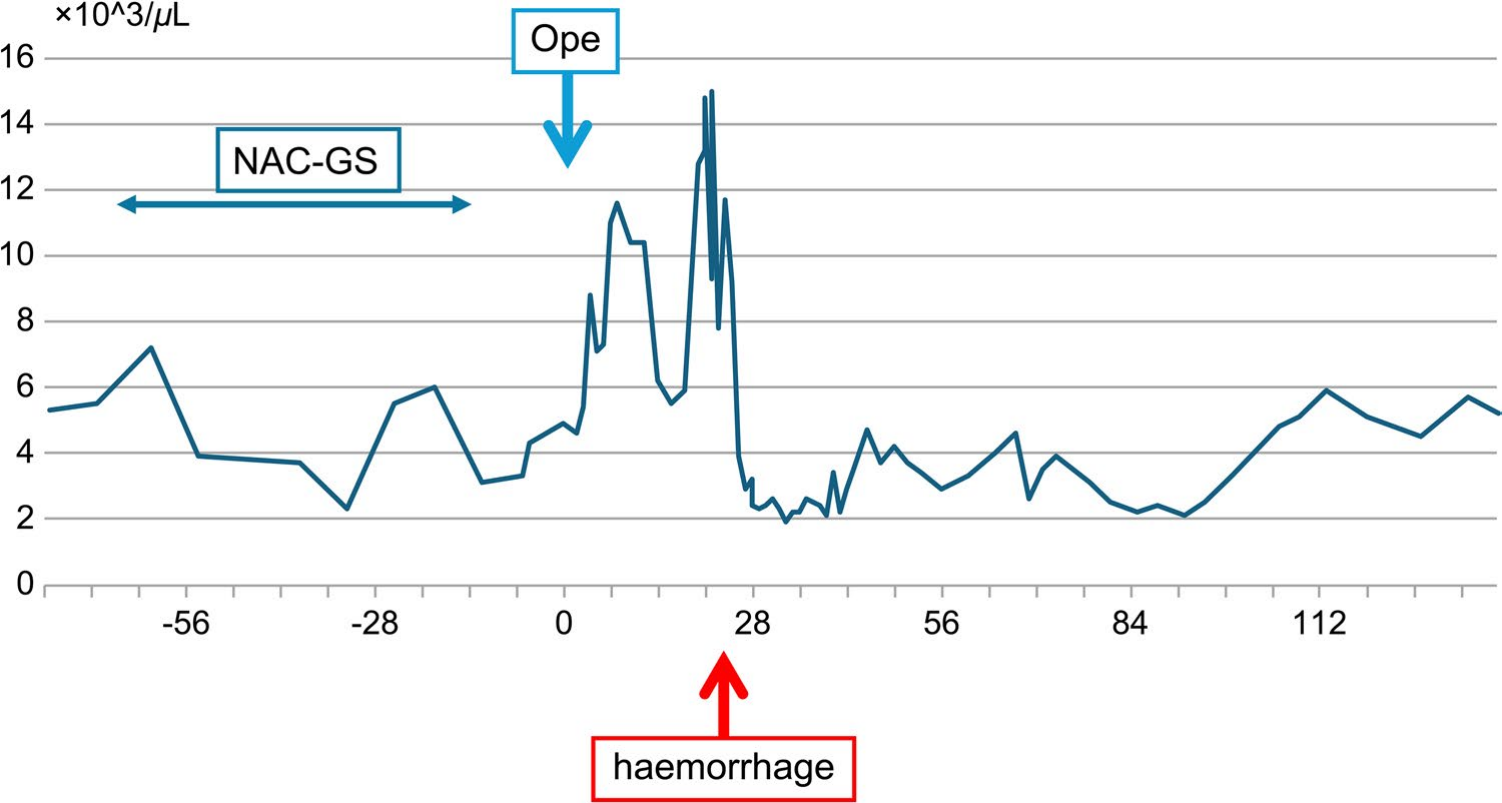
WBC. Grade III neutropenia during NAC-GS. *NAC-GS* neoadjuvant chemotherapy–gemcitabine plus S-1; *WBC* white blood cell; *Ope* surgical operation

The patient was diagnosed with resectable (R) pancreatic head cancer and was started on two courses of gemcitabine (GEM) plus S-1 (GS) as preoperative treatment (GEM at 1700 mg/body; S-1 at 120 mg/body; administered in a 2-weeks-on, 1-week-off schedule) 6 weeks after the initial diagnosis. Grade III neutropenia developed after the first dose of the second course, leading to a delay in treatment until after granulocyte colony-stimulating factor administration (Fig. 2). The patient had a mild coronavirus disease 2019 (COVID-19) infection during the waiting period. After the COVID-19 symptoms resolved, the second dose of the second GS course was administered at a reduced dose (GEM at 1500 mg/body; S-1 at 100 mg/body). No weight loss or performance status reduction was observed during chemotherapy.

Fifteen days after the last S-1 dose was administered, the patient underwent radical surgery. The pancreatic cancer was anterior and close to the mesentery of the colon, which led to the partial resection of the mesentery of the colon. Similarly, the portal vein was also proximal to the pancreas, necessitating its resection in a wedge-shaped fashion and suturing. The patient had constipation since the initiation of chemotherapy, and viscous feces were palpable in the transverse colon despite preoperative treatment. Following the standard procedure at our facility, sennoside 24 mg was administered orally prior to surgery to facilitate bowel movements. Specifically, the operative time and blood loss volume were 598 min and 1704 mL, respectively. Pathology results (Union of International Cancer Control 8th, Pancreas) showed that the tumor was 36 mm in diameter, invasive ductal carcinoma with IPMN, mod > wel, Ph, TS2, infiltrative type, int, INFb, Ly1a, V1a, Pn1a, mpd0, ypT3, ypCH0, ypDU1, ypS1, ypRP1, ypPV0, ypA0, ypPL0, ypOO0, ypPCM0, ypBCM0, ypDPM0, ypN1a(2/27), CY0, ypStageⅡB, and R0.

**Fig. 3:**
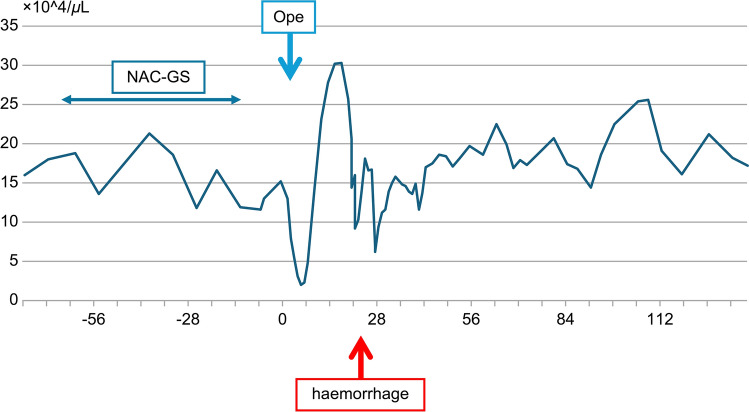
Platelets. Platelet count decreased immediately after surgery due to sepsis. *NAC-GS* neoadjuvant chemotherapy–gemcitabine plus S-1; *Ope* surgical operation

**Fig. 4:**
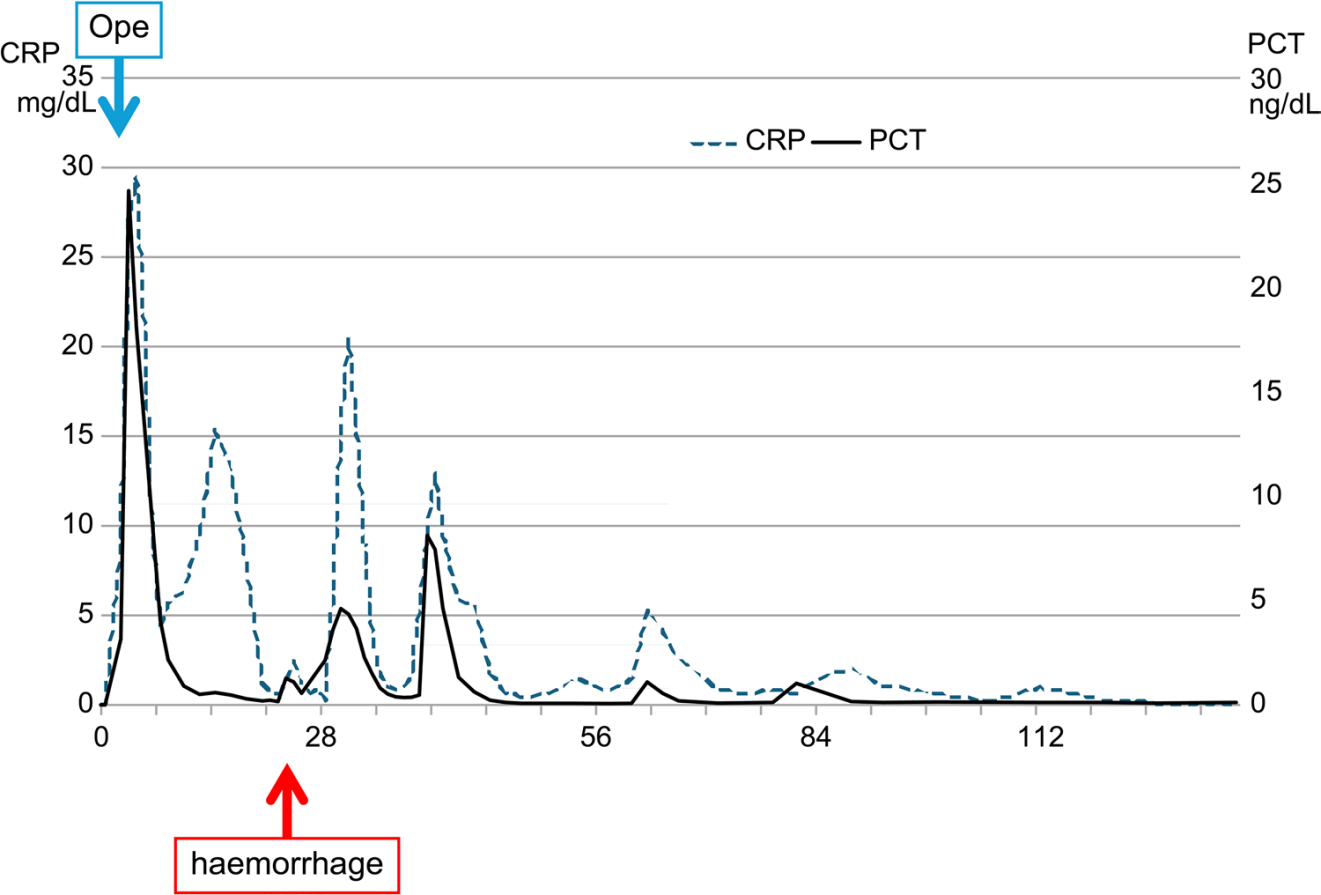
CRP and PCT. PCT was significantly elevated immediately after surgery. Findings suggestive of bacterial sepsis. *CRP* C-reactive protein; *NAC-GS* neoadjuvant chemotherapy–gemcitabine plus S-1; *PCT* procalcitonin; *Ope* surgical operation. **c** Fecal mass in the rectum. *POD* postoperative day

The preoperative and postoperative changes in white blood cell (WBC, Fig. 2), hemoglobin (Hb, Fig. S1), and platelet (PLT, Fig. 3) counts are shown in the figures. In addition, the postoperative changes in C-reactive protein (CRP) and procalcitonin (PCT) are shown in Fig. 4.

The patient’s blood pressure was hypotensive before the end of the surgery, and he was continued on hypertensive medications. After returning to the intensive care unit (ICU), the systolic blood pressure remained in the 60–80 mmHg range, even after increasing the noradrenaline to 0.3 gamma. Vasopressin was considered necessary to raise the blood pressure. The patient was conscious, showed no clinical symptoms, and had no complaint of abdominal pain. WBC counts were 4600 (postoperative day [POD]1), 5400 (POD2), and 8800 (POD3) cells/μL (Fig. 2), whereas CRP levels increased to 9.14 (POD1), 27.13 (POD2), and 29.46 (POD3) mg/dL (Fig. 4).

**Fig. 5:**
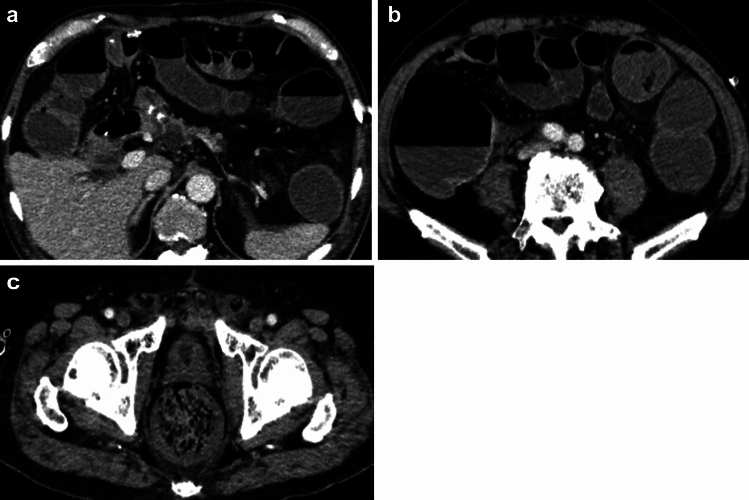
Fecal evidence of colonic dilatation and colitis (POD2). **a**, **b** Findings of colonic dilatation and thinning of the colon wall

Contrast-enhanced CT was performed on POD2 to evaluate for sepsis, potentially due to a pancreatic fistula or other causes (Fig. 5). No pancreatic fistula or intra-abdominal abscess was observed. After CT, the patient was patently excised after suppository placement, and a moderate fecal mass was egested. Soft stools were subsequently passed multiple times. The patient’s general condition improved rapidly after defecation, with a decrease in the need for pressure elevators over time. By the morning of POD4, no pressure elevators were needed. PCT levels improved rapidly after defecation, with values of 3.14 (POD1), 24.6 (POD2), and 17.98 (POD3) ng/dL (Fig. 4). Platelet count decreased to 130,000 (POD1), 54,000 (POD3), and 20,000 (POD5) cells/μL (Fig. 3), after which it gradually increased and normalized. Based on the disease course, we intraoperatively diagnosed the patient with fecal obstructive colitis that had progressed to septicemia.

Cefmetazole was intraoperatively administered as a prophylactic antibiotic. The culture results of the drain fluid from POD2, as well as blood cultures (arterial and venous blood), were negative. In addition, the posterior segment of the bile duct branched early, and a bile duct stent was placed. Bile cultures from this site revealed *Enterococcus faecalis* (2+) and *Klebsiella aerogenes* (1+).

Following this course, watery stools were observed; however, stool cultures and *Clostridioides difficile* toxin tests ruled out methicillin-resistant *Staphylococcus aureus* enterocolitis or *Clostridium enterocolitis*. No pancreatic or biliary fistulas were observed, and the drainage tube was removed on POD7. However, anemia progressed from POD17. Upper gastrointestinal endoscopy revealed a blood flow disturbance in the gastric mucosa. The stomach was filled with a large amount of blood; however, the bleeding point could not be identified. On the next day (POD22), endoscopy of the small intestine revealed bleeding from the bile duct–jejunal anastomosis (Fig. S2). An emergency angiography was performed, revealing an aneurysm at the treated portion of the cystic artery. The aneurysm had ruptured in the jejunal limb, causing bleeding into the jejunum. In addition, the gallbladder artery originates from a branch of the posterior regional branch. The root of the cystic artery was embolized with a microcoil, and hemostasis was achieved. However, the patient presented with another episode of bleeding on POD26, and angiography was repeated. This time, a pseudoaneurysm had formed at the cholecystic artery processing site, and bleeding recurred. Part of the previous coil had fallen into the gastrointestinal tract. Therefore, the cystic artery was embolized from the cystic artery to the posterior regional branch of the hepatic artery (Fig. S3).

**Fig. 6:**
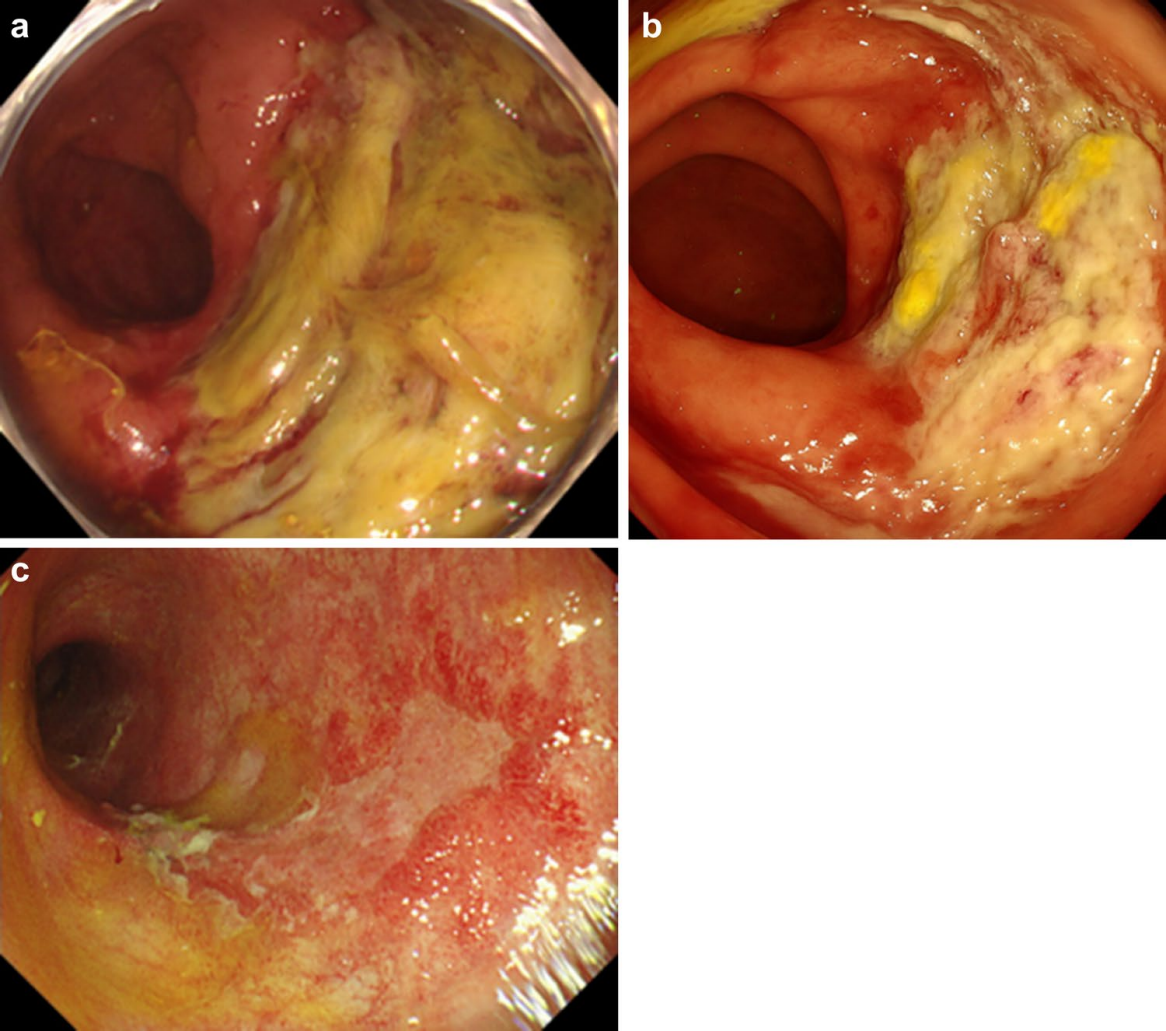
Colonoscopy findings. **a** POD35, Part of multiple ulcers, map-like ulcer. **b** POD60, CMV-negative in the blood, but blood loss and diarrhea after feeding. **c** POD97, ulcer became scarred, and some healed ulcer areas showed stenosis. *POD* postoperative day; *CMV* cytomegalovirus

**Fig. 7:**
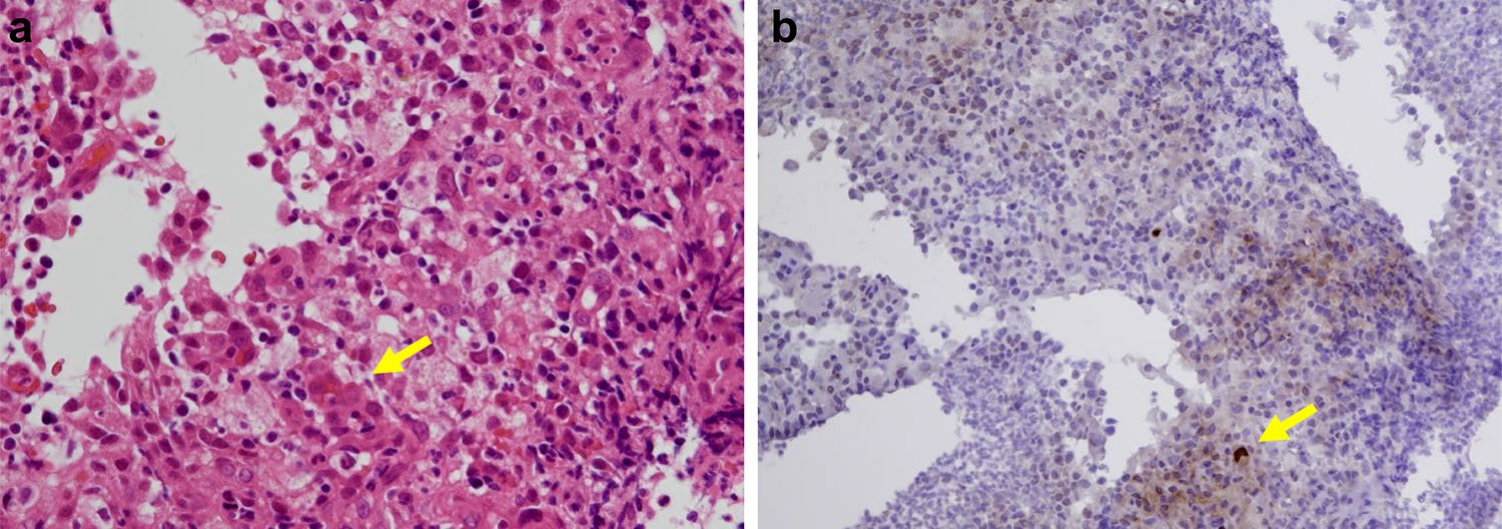
POD35, colon biopsy pathology. **a** H&E (40 ×), inflammatory cellular infiltrate, including neutrophils. No specific inflammatory findings. Suspected CMV infection (arrow). **b** CMV antigen staining (20 ×), scattered cells positive for CMV (arrow). *POD* postoperative day; *CMV* cytomegalovirus; *H&E* hematoxylin and eosin

While his procedure controlled the bleeding from the same area, the patient continued to experience diarrhea and intermittent episodes of bleeding. CT showed no findings suggestive of gastrointestinal bleeding from the aneurysm. Colonoscopy at POD35 revealed multiple ulcerations and hemorrhage. Scattered map-like ulcers were accompanied by a mossy white appearance (Fig. 6a). The pathological diagnosis from the colon biopsy revealed mainly nonspecific inflammatory findings (Fig. 7a); however, immunostaining showed scattered CMV-positive cells (Fig. 7b). A diagnosis of CMV enteritis was made based on the characteristic endoscopic findings, biopsy results, and positive blood anti-CMV antibody results. Treatment with ganciclovir (180 mg/day) resulted in a mild relief of colitis in approximately 2 weeks (Fig. 6b). Colonoscopy performed on POD97 showed healing of the ulcer (Fig. 6c). The patient was discharged on POD136, although it took time for his nutritional condition to improve and for rehabilitation to progress. Notably, he has survived for approximately 6 months without recurrence, despite not receiving postoperative adjuvant chemotherapy.

## Discussion

Obstructive colitis is reportedly caused by cancer in approximately 90% of cases, making it a relatively common occurrence. The symptoms include abdominal pain, abdominal distention, and fever; however, no characteristic findings exist [6, 7]. Fecal obstructive colitis has also been reported to occur when hard fecal stones or large volumes of fecal material accumulate in the intestinal tract, obstructing the passage of intestinal contents. Male age, aging, laparotomy, and surgery lasting > 3 h are risk factors for postoperative fecal ileus in gastrointestinal surgery, such as colon resection [8–11]. When fecal ileus progresses to necrosis of the colon, determining the progression from abdominal or CT findings is challenging. In the present case, the patient was aware of a tendency toward constipation during preoperative chemotherapy. He was treated with preoperative sennoside to control defecation, and the same procedure was used in this case. However, a fecal mass was palpable in the transverse colon intraoperatively, and stool clearance was poor. Resection of the lesion required treatment of the mesocolon, along with wedge-shaped resection and portal vein reconstruction. These procedures may have further interfered with colonic motility. Intraoperative colonic pressure increased, leading to bacterial translocation, which was believed to contribute to the decrease in blood pressure near the end of surgery [12]. This case involved combined resection of the portal vein and resection of the mesocolon; thus, mechanical bowel preparation should have been performed prior to surgery. The postoperative course in the ICU showed sepsis progression, although without clinical manifestations. CT findings on POD2 showed that fecal colon dilation was an unusual finding but not a definite cause of the disease. However, we considered it a contributing factor and encouraged defecation via excision, which resulted in the induction of a large amount of defecation and significant improvement in the patient’s condition. Frequently responding to minor findings during the perioperative period is necessary in highly invasive surgeries. In fact, a surgical review of highly skilled hepatobiliary and pancreatic centers in Japan reported a low mortality rate, despite a high complication rate in centers with a large number of cases [13]. One possible explanation for this phenomenon is that more experienced facilities may achieve higher survival rates due to their proactive approach in examining and treating complications that are easily missed, as demonstrated in this case.

Resection is performed after preoperative treatment in resectable (R) pancreatic cancer cases, and adjuvant chemotherapy is the standard approach postoperatively [4, 5]. These procedures can cause pancytopenia, potentially leading to a decrease in the number of pancytopenic cells. In addition, many gastrointestinal symptoms, such as anorexia, diarrhea, and constipation, may occur and are feared to be caused by decreased immunity. Perioperative complications, such as pancreatic fistulas, are frequent, and radical resection is highly invasive and associated with high mortality rates. Grade III neutropenia was observed during preoperative treatment, and the final dose was administered at a reduced level. COVID-19 infection was also detected during the same period. The pancytopenia and constipation that occurred during preoperative treatment may have affected the intraoperative progression of colitis obliterans. Therefore, adequate preoperative defecation control is necessary for patients scheduled for combined portal vein resection or extensive mesocolon resection.

In the present case, the patient experienced bleeding from an aneurysm, which is one of the most important complications of pancreaticoduodenectomy. It is believed to have been caused by a minor suture failure during the treatment of the cystic artery and bile duct–jejunal anastomosis. This can be a fatal complication that requires massive blood transfusions and results in an extremely poor general condition. CMV enteritis was confirmed after a life-saving hemorrhage. The endoscopic findings of CMV enteritis are relatively characteristic; however, clinical manifestations such as bloody stools are absent [14–16]. In addition, the positive rate of CMV antibodies in the blood was low. In the present case, the patient had obstructive colitis immediately after surgery, with elevated CRP and procalcitonin levels, which rapidly improved after defecation; however, only the CRP level was elevated again. Colonoscopy led to the diagnosis of CMV enteritis. Although the patient had a hemorrhage following the gastrointestinal tract perforation by an aneurysm, CT and clinical signs were negative for intra-abdominal suture insufficiency or rebleeding. Since the possibility of the obstructive colitis worsening again could not be ruled out, an endoscopic examination was performed. The multiple ulcers and moss-like lesions observed via endoscopy suggested CMV enteritis, and a colon biopsy was performed. CMV enteritis is uncommon after gastrointestinal surgery. Thus, this case highlights the importance of endoscopic procedures for the treatment of postoperative hematochezia. The combination of preoperative chemotherapy, massive bleeding, and colitis obliterans is an important factor in CMV enteritis development. Mucosal destruction due to ischemic colitis or irritable bowel syndrome is believed to lead to local immune suppression and CMV activation [17]. These conditions can occur during the perioperative period of radical pancreatic cancer surgery, and vigilance is required.

The treatment approach for CMV enteritis involved the intravenous administration of ganciclovir. In addition, the CMV antigen test result was positive at POD35, which turned negative at POD42 and subsequently remained negative. Ganciclovir administration is generally recommended for approximately 2–3 weeks [18, 19]. Its administration was discontinued on POD46 since the CMV antigen test result was negative. However, ganciclovir was readministered owing to the recurrence of symptoms, such as fever and bloody stools, resulting in symptom improvement. On POD60, colonoscopy revealed that the ulcers characteristic of CMV enteritis had slightly decreased in size but remained multiple with residual white patches and bleeding (Fig. 6b). However, a subsequent biopsy did not reveal CMV-infected cells. Ulcer scarring was confirmed on POD97 (Fig. 6c). Colonoscopy is not considered mandatory in CMV enteritis if symptoms improve and the viral load decreases [20]. In the present case, colonoscopy was beneficial for assessing symptom improvement and treatment outcomes in addition to CMV antigen confirmation. Various causes, such as hematemesis, can be considered for perioperative symptoms. As in the present case, the causes and treatment targets of complications may differ over time. Therefore, appropriate testing and treatment should be initiated before the occurrence of systemic deterioration.

The patient in this case presented with mild COVID-19 infection during preoperative chemotherapy. Typically, following COVID infection, long-term immune dysfunction [21] or the coexistence of excessive inflammation and immunosuppression can occur, which may contribute to the risk of other diseases through mechanisms such as infection recurrence, induction of autoimmune responses, and changes in tumor-related pathways [22]. In addition, it has been reported that high frequencies of CMV reactivation were observed in the blood of patients with severe COVID [23]. Contrastingly, it is considered difficult to conclude that mild COVID infection directly contributes to CMV activation in cases like the present one.

In conclusion, the postoperative mortality rate for pancreatic cancer remains higher than that for other malignant diseases. Close postoperative management is essential to reduce mortality after this type of surgery. No reports exist on the perioperative effects of bowel obstruction or CMV enteritis caused by fecal matter in the context of pancreatic cancer. Colonic, mesenteric, and portal vein resections are commonly performed in patients with advanced pancreatic cancers. Preoperative chemotherapy is also increasingly being administered. Various effects may occur over time with a multidisciplinary treatment strategy that combines chemotherapy with highly invasive surgeries. Symptoms such as dysuria and bloody stools should not be overlooked, and CT scans and colonoscopies should be performed, as necessary, to identify the cause of the disease and prevent the occurrence of potentially fatal adverse events and complications. This case illustrates the importance of perioperative defecation control during highly invasive surgery with preoperative treatment and colonoscopy at the time of hemorrhage.

## Supplementary Information

Below is the link to the electronic supplementary material.

Supplementary file1 (DOCX 312 KB)
