# Supplementary material for: Fecal colitis obliterans and cytomegalovirus enteritis after pancreaticoduodenectomy for resectable pancreatic cancer
Source: Clin J Gastroenterol. 2025 Sep 12;18(6):1118–25. doi: 10.1007/s12328-025-02219-7 (PMC12630215; doi:10.1007/s12328-025-02219-7)
Supplement: Supplementary file 1 — Supplementary file1 (DOCX 312 KB) [file 12328_2025_2219_MOESM1_ESM.docx]

**Electronic Supplementary Materials**


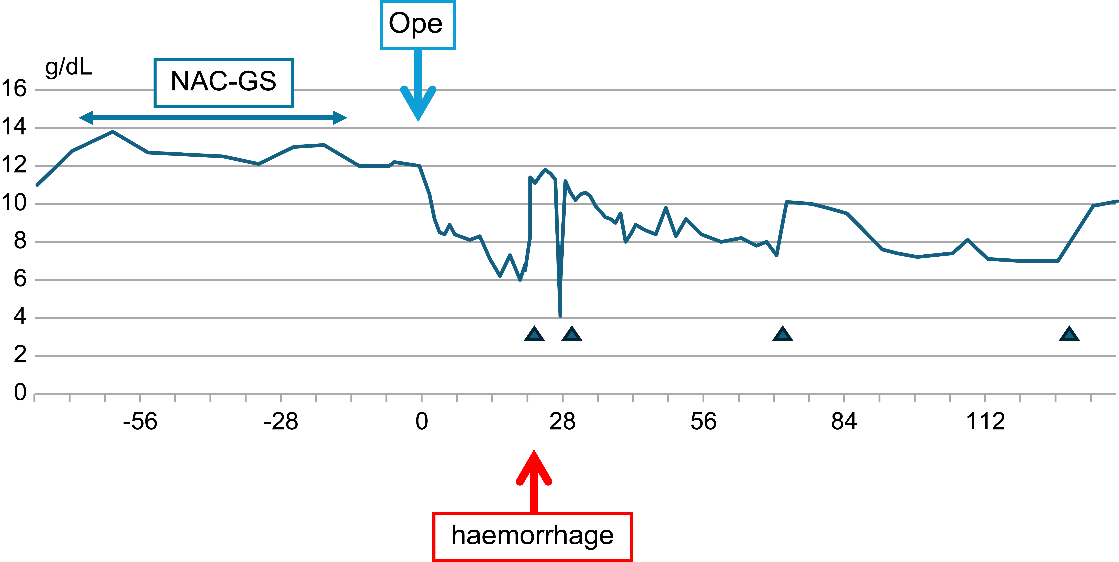


**Fig. S1** Hb

Bleeding from an aneurysm on PODs21–26

RBC transfusion is marked with △.

RBC, red blood cell; POD, postoperative day; Hb, hemoglobin; NAC-GS, neoadjuvant chemotherapy-gemcitabine plus S-1; Ope, Surgical Operation


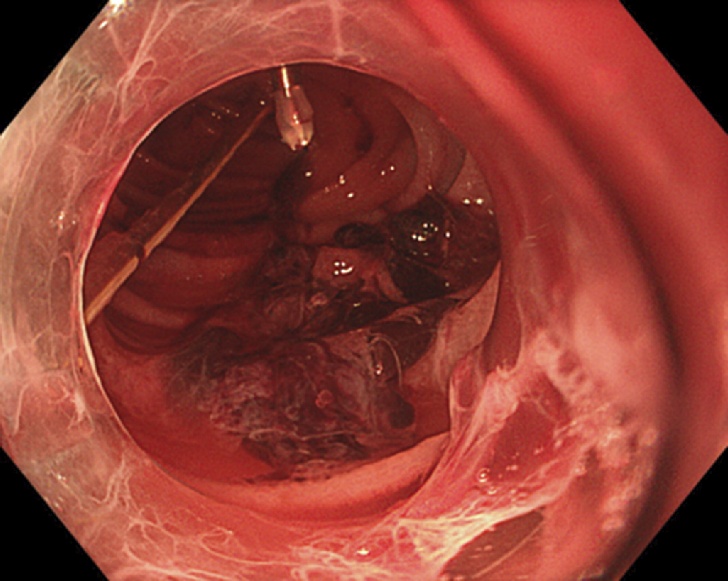


**Fig.S2** Small bowel endoscopy (POD22)

A bile duct stent implanted in the posterior region is visible.

A marking clip is placed near the bleeding point.

POD, postoperative day


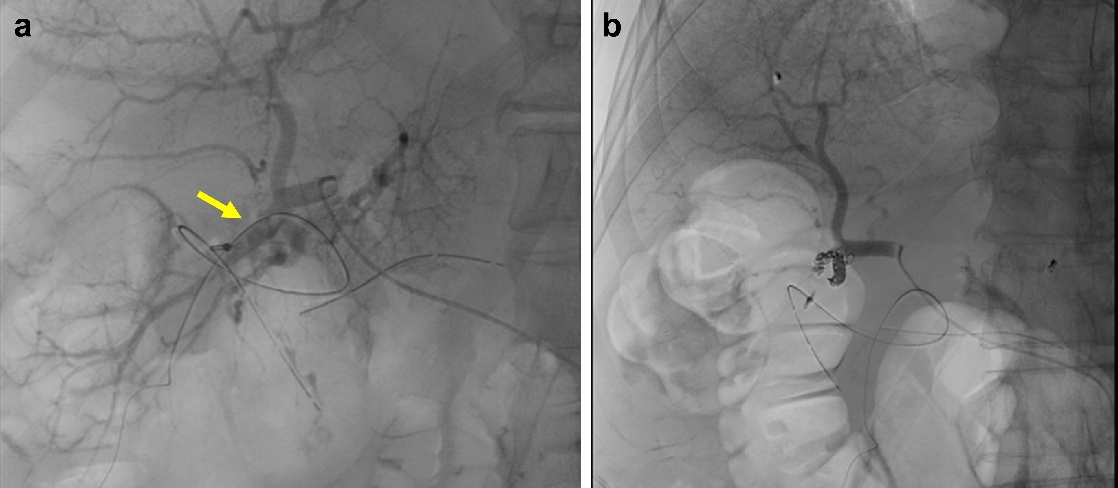


**Fig. S3** Angiography findings

a: Extravasation into the intestinal canal from the ileocecal artery

b: Hemostasis using a coil
